# Hermetic Storage as a Chemical-Free Alternative for Millet Preservation in Niger

**DOI:** 10.3390/insects16090917

**Published:** 2025-09-02

**Authors:** Habibou Yahaya Dan Bawa, Ibrahim Boukary Baoua, Mahamane Moctar Rabé, Dieudonne Baributsa

**Affiliations:** 1 Faculté d’Agronomie et des Sciences de l’Environnement, Université Dan Dicko Dankoulodo de Maradi, Maradi BP 465, Niger; habibyahaya34@gmail.com (H.Y.D.B.); baoua.ibrahim@gmail.com (I.B.B.); 2 Départment de Sociologie et Economie Rurale, Université de Tahoua, Tahoua BP 255, Niger; mocnad1@gmail.com; 3 Department of Entomology, Purdue University, West Lafayette, IN 47907, USA

**Keywords:** *Trogoderma granarium*, grain storage, pesticides, PICS bags, Sahel

## Abstract

Millet (*Pennisetum glaucum* (L.) R. Br.) is the most widely consumed cereal in Niger. Although its production lasts six months, consumption is year-round, requiring effective and safe storage solutions. Post-harvest losses of millet can reach up to 17% after several months of storage. Chemical pesticides are commonly used in large warehouses, but concerns over misuse and health risks have driven interest in safer alternatives. This study assessed the effectiveness of traditional and improved storage methods in controlling *Trogoderma granarium* Everts, an emerging pest of millet in large warehouse facilities. Three storage methods were tested: (i) a hermetic Purdue Improved Crop Storage (PICS) bag; (ii) chemical pesticide Phostoxin; and (iii) an untreated ordinary polypropylene bag as a control. Naturally infested millet obtained from a large warehouse was assessed for each treatment at the start and end of a six-month storage period. Two insect pests were identified, *T. granarium* and *Tribolium* sp. Initial infestation levels were 60 larvae and 0.47 adults per 500 g. After six months, the control showed a 1.7-fold increase in *T. granarium* larvae, a 2.96-fold increase in its adults, and a 3.19-fold increase in *Tribolium* sp. adults, resulting in 19.15% weight loss. In contrast, PICS and Phostoxin treatments maintained initial pest levels with no weight loss. These results suggest that hermetic storage is a safe, effective, and chemical-free alternative for preserving millet in large warehouses.

## Simple Summary

Millet is the most consumed cereal in Niger, but its seasonal production and year-round demand create a strong need for safe long-term storage. Although pesticides are commonly used in warehouses, concerns over misuse and safety highlight the need for safer alternatives. This study evaluated the effectiveness of (i) hermetic bags, (ii) pesticide (Phostoxin or aluminum phosphide), and (iii) regular polypropylene bags without chemicals in controlling *Trogoderma granarium*, an emerging pest of stored millet in large warehouses. Infested millet from the national grain reserve was stored using these treatments and analyzed at the start and after six months. The control treatment showed significant increases in pest numbers, resulting in a 19.15% loss of grain weight. In contrast, both hermetic bags and Phostoxin treatments prevented further infestation. Overall, hermetic bags proved to be an effective, safer, and chemical-free storage option.

## 1. Introduction

In many African countries, cereals such as millet, sorghum, wheat, maize, and rice form the foundation of the rural diet, especially among low-income populations [1,2,3]. These cereals play a critical role in ensuring food and nutrition security across sub-Saharan Africa [4]. Among them, millet is particularly important in arid and semi-arid areas, including the Sahel, where it serves as a major source of energy [5,6,7].

In Niger, millet is an important cereal. Over the past several decades, millet production in Niger has experienced significant growth in both cultivated area and yields. For instance, production increased from 776,000 tons on 1,640,100 hectares in 1961 to 581,300 tons on 1,692,900 hectares in 1975, and then to 3,321,753 tons on 7,358,247 hectares in 2014 [8]. In 2024, millet production was recorded at 3,161,868 tons over 6,736,592 hectares [9], representing an increase of 307% in output and 311% in cultivated area compared to 1961. Although production has increased significantly over the years, supply remains limited due to various factors, including post-harvest storage losses.

In the dry Sahelian zone, post-harvest storage is essential, as it bridges the gap between the annual harvest and continuous household consumption [3,10]. However, achieving food self-sufficiency is often constrained by low production and poor post-harvest management practices [6]. Insects are one of the major challenges to effective grain storage. Millet stocks are particularly vulnerable to numerous pests including *Corcyra cephalonica* (Stainton), *Tribolium castaneum* (Herbst, 1797), *Tribolium confusum* Jacquelin du Val, *Sitotroga cerealella* (Olivier), *Oryzeaphilus mercator* (Fauvel), *Lasioderma serricorne* (Fabricius), *Rhyzopertha dominica* (Fabricius), *Sitophilus oryzae* (Linnaeus), *Ephestia cautella* (Walker), *Cryptolestes ferrugineus* (Stephens), and *Trogoderma granarium* Everts [11,12,13,14,15]. In Niger, a study identified *C. cephalonica* and *Tribolium* spp. as key pests, causing an estimated post-harvest loss of 17.1% [16].

*Trogoderma granarium*, also known as the khapra beetle, is a destructive pest of stored products. This pest is considered one of the most serious threats to stored cereals and legumes globally, especially in tropical and subtropical regions [17,18]. Native to the Indian subcontinent, *T. granarium* has spread to parts of Africa, Asia, Europe, and South America [19]. It is a quarantine pest in many parts of the Western Hemisphere, continental Europe, and Asia [20]. It has been found in groundnut stocks in the Maradi and Zinder regions of Niger [21] and more recently in large grain warehouses in both regions [22]. These large storage facilities comprise government food reserves or stocks held by development agencies to support emergency responses during food shortages and crises.

The larva of *T. granarium* feeds aggressively on stored grains, often leaving behind only the outer pericarp. Its presence contaminates food products with exuviae and excreta, compromising food quality. The pest is particularly difficult to control because its larvae can hide in crevices and enter diapause for extended periods, even years [20,23]. Furthermore, repeated use of synthetic pesticides has led to increasing insecticide resistance, with *T. granarium* showing high tolerance to several chemical treatments, temperature, and growth regulators [11,24,25,26]. Despite this, large warehouse managers and farmers continue to rely on unapproved chemical treatments for grain protection [27,28,29], highlighting the urgent need for safer and more sustainable storage solutions.

Significant improvements in post-harvest grain protection have been achieved in the Sahelian region due to the introduction and widespread adoption of hermetic storage technologies such as the Purdue Improved Crop Storage (PICS) bag [30,31]. Farmers and large-scale users, such as the Office des Produits Vivriers du Niger (OPVN), have used the technology for cowpea storage [32]. A study in Niger comparing five commonly used storage technologies in sub-Saharan Africa confirmed that PICS bags were effective in protecting stored cowpeas and maize [29,33,34]. No study has investigated the use of hermetic bags for storing millet. In this context, the current study was conducted to evaluate the effectiveness of hermetic storage against *T. granarium* on grain stored in large warehouses in south-central Niger.

Though *T. granarium* has recently been reported on millet in large warehouses, data on its impact and control remain limited. Though PICS bags are effective in storing cereals, their efficacy against *T. granarium* on millet has not been studied in the Sahel and particularly in Niger. This study addresses this gap by evaluating the damage caused by this pest and testing whether hermetic storage can minimize losses. Beyond reducing pest damage, hermetic storage offers significant food safety benefits by eliminating pesticide residues and improves worker safety by reducing exposure to hazardous chemicals commonly used in warehouses. The study thus contributes to both improved grain protection and safer storage practices in Niger.

## 2. Materials and Methods

The study was conducted in the laboratory of the Biodiversity Center at the University of Maradi (UDDM) in Niger from 25 August 2023, to 25 February 2024. The experiment compared three post-harvest storage methods: (i) PICS hermetic bag with two liners fitted into one woven bag [29]; (ii) Fumigation with Phostoxin or aluminum phosphide (1 tablet per bag), a pesticide containing aluminum phosphide applied in millet stored in a standard polypropylene bag lined with a 50 μm thick plastic liner (positive control); (iii) Ordinary polypropylene bag without pesticide, used as a negative control. This study used 50 kg bags, the standard size commonly employed in warehouses for grain storage. The PICS bags were obtained from the manufacturer, Lela Agro Industries (Kano, Nigeria), while the Phostoxin tablets, manufactured by Degesch America Inc. (Weyers Cave, VA, USA) and regular PP bags were purchased from local input dealers’ shops.

Millet grains used in the experiment were obtained from the National Food Security Agency, Office des Produits Vivriers du Niger (OPVN). In the Sahel, cowpeas in warehouses are treated with chemicals, but millet is not. The spread of *T. granarium* highlights the need for more effective control methods. The millet used in this study had been stored untreated for three years and was already naturally infested. All millet was thoroughly mixed to homogenize the levels of insect pest infestation. Each treatment consisted of four replicates, with 40 kg of millet per bag, resulting in a total of 12 bags. Once filled, the bags were stored inside the laboratory under ambient temperature and relative humidity conditions for the duration of the experiment.

Infestation levels were evaluated at both the beginning and end of the experiment using twelve 500 g samples (three samples per replicate). Each 500 g sample was manually sorted, and the density of pests was determined by counting all live larvae and adult insects present. From each of these, three 100-grain sub-samples (*n* = 36 per treatment) were analyzed to determine the total weight, number of damaged kernels, and the weight of damaged kernels and impurities. The level of impurities was assessed using 500 g samples, which were sorted to separate non-edible organic and inorganic materials. Each fraction was then weighed, and its proportion calculated relative to the original sample. Insect mortality, grain damage and weight loss were calculated using the following formulas

$$\%\;mortality=\frac{\left(Initial\;pest\;density-Density\;in\;treatment\;after\;6\;months\right)}{Initial\;pest\;density}\times 100$$

$$\%\;infested\;grains=\frac{Number\;of\;infested\;grains}{Total\;number\;of\;grains}\times 100$$

$$\%\;weight\;loss=\frac{\left(Final\;weight-Initial\;weight\right)}{Initial\;weight}\times 100$$

To monitor environmental conditions, EL-USB-2 dataloggers (Lascar, Whiteparish, Wiltshire, UK) were placed both inside the experimental room and within one bag per treatment. These devices recorded temperature and relative humidity every hour throughout the experiment. For the PICS hermetic bags, oxygen (O_2_) and carbon dioxide (CO_2_) concentrations were measured at the start of the experiment, as well as at the end of the first and second months (between 27 August and 27 October 2023). These gas levels were monitored using a Mocon PAC Check Model 325 headspace analyzer (Mocon, Minneapolis, MN, USA).

Data analysis was conducted using the Statistical Package for the Social Sciences (SPSS) version 22.0. Analysis of variance (ANOVA) was used to assess treatment effects. When ANOVA results were significant, means were compared using the Student-Newman-Keuls test at a 5% significance level. When the data did not follow a normal distribution, the Kruskal-Wallis test was used to compare group means.

## 3. Results

**Figure 1:**
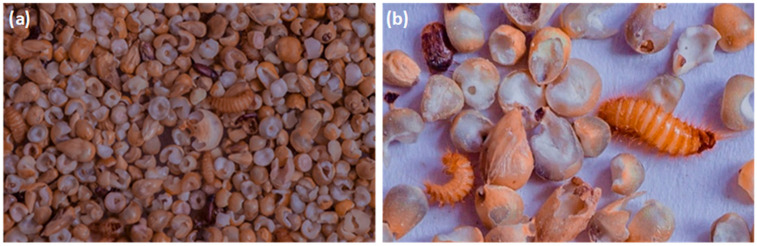
Millet (**a**) infested by *Trogoderma granarium* and *Tribolium* sp., (**b**) damaged by larva of *T. granarium* in control after six months of storage.

The relative humidity ranged from 21.16% to 67.73%, and the average daily temperature recorded was between 26.18 °C and 37.89 °C during the experimental period. In the PICS bag, the oxygen concentration varied over time. At the closing of the bags, an average oxygen concentration of 20.73 ± 0.34% was noted but decreased to 16.98 ± 1.42% after one month and rose to 18.96 ± 0.69% after two months. The CO_2_ levels were undetectable at the beginning of the experiment but increased to 5.08 ± 1.99% after one month and then decreased to 2.16 ± 0.78% after the second month. Collected millet samples revealed the presence of two insect pest species: *T. granarium* and *Tribolium* sp. (Table 1, Figure 1).

The insect population increased in the control while it decreased in the treatments. After 6 months of storage, larvae and pupae of *T. granarium* decreased by 98.2% in the Phostoxin treatment and by 98.8% in the PICS bag treatment (Table 1). In contrast, in the control, the population of larvae and pupae was 1.7 times higher than that of the initial level. The Adult *T. granarium* decreased by 39.3% and 71.4% in the Phostoxin and PICS bag treatments, respectively, compared to the initial infestation level. Conversely, in the control, it increased by 2.96 times compared to the initial. For *Tribolium* sp., the insect population declined to 0.25 and 0.08 adults per 500 g in the Phostoxin and PICS bag treatments, respectively, but increased 3.2-fold in the control. The initial impurity content was 4.08%. After six months, impurity levels remained stable in the Phostoxin and PICS treatments, while the control showed a 4.95 percentage points increase.

The number of kernels damaged by insect pests varied over time (*p* < 0.05) (Table 2). This ratio increased by 3.17% in the control treatment, whereas in the Phostoxin and the PICS bag treatments, it remained similar to levels observed at the beginning of the experiment. At the end of the experiment, the weight of 100 seeds varied by treatment (*p* < 0.001) (Table 2). After 6 months of storage in the PICS bag and Phostoxin treatments, the weight of 100 seeds remained comparable to the initial values at the start of the experiment. In the control treatment, however, the weight of 100 seeds after 6 months was 19.15% lower compared to levels measured at the beginning of the study.

## 4. Discussion

This study was conducted under ambient Sahelian conditions, which reflect typical environments for grain storage by farmers, traders, and warehouses. Although rarely reported in smallholder settings in Niger, *T. granarium* is a well-known pest of stored cereals and legumes across Sahelian Africa [12,22,35]. Under Sahelian conditions, millet was found to be susceptible to infestation by *T. granarium* and *Tribolium* sp. The detection of *T. granarium* in large warehouses is consistent with its known association with centralized storage systems and international grain flows. As a polyphagous, quarantine pest with long-lived diapausing larvae, *T. granarium* presents persistent risks in storage environments, particularly where cracks and crevices allow for hidden infestations [36,37,38,39]. In contrast, smallholder storage in Niger typically reports pests such as *C. cephalonica*, *S. cerealella*, and *Sitophilus* spp. [12,16,40,41,42]. *Tribolium* sp., the second pest identified, is commonly observed on cereals but is generally considered a secondary pest of limited economic importance, as it primarily feeds on pre-damaged seeds [43,44].

In untreated control bags, populations of *T. granarium* and *Tribolium* sp. increased significantly over a six-month period. These infestations contributed to an increase in damaged grains, leading to greater weight loss. Although these losses are substantial, they are lower than those typically reported for cowpea storage in the Sahel, which often exceeds 35–70% loss [29,45,46]. Nevertheless, the findings confirm that millet is susceptible to significant deterioration when stored without effective protection, particularly in large-scale facilities where pest introductions and spread are more challenging to control. Comparable studies have reported substantial crop losses due to storage pests. *Trogoderma granarium* has been shown to cause weight losses ranging from 2% to 70%, depending on the type of stored product [47,48]. In India, losses of up to 61% due to *T. granarium* were observed on untreated millet after six months of storage [49].

Phostoxin fumigation proved highly effective, significantly reducing the density of *T. granarium* larvae, pupae, and adults. Similarly, *Tribolium* sp. populations were reduced. These results confirm the fumigant’s potency when applied under controlled conditions, especially with a tight containment system using a waterproof plastic liner. Studies have shown that Phostoxin is effective in controlling *T. granarium* in stored grains, including millet [12,22]. However, the use of Phostoxin presents serious safety concerns. It is toxic to humans and animals and may leave harmful chemical residues in the grain treated [50,51,52,53,54]. Additional concerns include workers’ safety (applicators) and the development of insect resistance to this pesticide due to repeated applications over long storage durations [52,55,56,57,58,59], a common practice in these large food security warehouses.

PICS bags offered a safer, chemical-free, and equally effective alternative. Oxygen depletion and CO_2_ buildup inside the bags created modified atmospheres that killed insects through suffocation and desiccation [60,61]. After six months, the millet stored in PICS bags retained its physical quality, with no significant increases in seed damage or weight loss. The PICS technology has been widely demonstrated to protect a variety of crops [62,63]. In addition to eliminating pest threats without the use of chemicals, the PICS bag provides economic and financial benefits for smallholder farmers and grain traders [31,64]. Beyond pest control, PICS bags support improved food safety by avoiding pesticide residues and enhancing worker safety by eliminating exposure to harmful fumigants. The broad efficacy and cost-effectiveness of hermetic bags compared to pesticides across various crops make it a highly suitable option as a safe and sustainable post-harvest management approach in the Sahel [29,65,66]. Scaling the use of PICS bags for millet storage in large warehouses will not be a challenge as the same technology has been used by food security agencies (e.g., OPVN) for cowpea storage [32].

## 5. Conclusions

This study confirms that millet stored under ambient Sahelian conditions is susceptible to infestation by *T. granarium* and *Tribolium* sp., particularly in large-scale storage environments. In the absence of control measures, pest populations increased significantly over six months, resulting in measurable losses in grain quality, including increased seed damage and weight loss. Both Phostoxin fumigation and hermetic storage using PICS bags were effective in suppressing pest populations and preserving millet quality. However, Phostoxin poses considerable risks to human health and the environment due to misuse and overuse, chemical residues, and the potential for insect resistance. In contrast, PICS bags maintained grain quality without the use of synthetic chemicals, offering a safer and more sustainable alternative. The findings underscore the potential of hermetic storage as a practical and scalable solution for improving post-harvest grain protection in large-scale warehouses in the Sahel. PICS technology not only reduces losses from insect pests but also enhances food safety and reduces occupational exposure to hazardous pesticides.

PICS hermetic bags should be promoted as a safe and effective method for storing millet, particularly in large warehouses, including those used by food security agencies. The use of chemical fumigants, such as Phostoxin, should be restricted to well-managed environments with appropriate safety measures to minimize health risks and the development of resistance. Strengthening the capacity of farmers, traders, and warehouse managers and workers through targeted training will support the adoption of safer storage practices. Ultimately, further research is necessary to assess the long-term effect of hermetic storage on the life cycle of *T. granarium*, particularly the larvae that enter diapause for extended periods. Additionally, understanding policies that incentivize millet value chain actors to use chemical-free storage methods is crucial for enhancing food safety and security.

## Figures and Tables

**Table 1:** Infestation and proportion of impurities per 500 g of naturally infested millet stored for six months.

Sampling Period	Treatment	*n*	Sample of 500 g of Millet			
			Weight of Impurities (g)	Number of *T. granarium* Larvae and Pupae	Number of Adults of *T. granarium*	Number of Adults of *Tribolium* sp.
Initial (0 month)		36	20.40 ± 0.88 b *	60.67 ± 5.61 b	0.28 ± 0.11 b	0.47 ± 0.22 a
After 6 months	Control	12	25.35 ± 1.79 a	103.17 ± 17.89 a	0.83 ± 0.35 a	1.50 ± 0.97 a
	Phostoxin	12	20.47 ± 0.73 b	1.08 ± 0.48 c	0.17 ± 0.11 b	0.25 ± 0.13 a
	PICS Bag	12	20.34 ± 0.61 b	0.75 ± 0.25 c	0.08 ± 0.08 b	0.08 ± 0.08 a
	ANOVA		F = 3.622; *p* < 0.005	F = 26.571; *p* < 0.001	F = 2.831; *p* < 0.05	F = 1.804; *p* > 0.05

* All data are means ± standard error of means (SEM). Means within the same column followed by the same letter are not significantly different (*p* > 0.05).

**Table 2:** Weight and damage of insect pests per 100 seeds of naturally infested millet stored for six months.

Sampling Period	Treatment	*n*	Sample of 100 Millet Seeds	
			Weight (g)	Number of Damaged Seeds
Initial (0 month)		108	0.94 ± 0.13 a *	15.3 ± 4.9 b
After 6 months	Control	36	0.76 ± 0.13 b	18.5 ± 5.9 a
	Phostoxin	36	0.96 ± 0.13 a	15.6 ± 4.9 b
	PICS bags	36	0.97 ± 0.11 a	16.1 ± 4.5 b
			F = 21.912; *p* < 0.001 ^†^	H = 8.86; *p* < 0.05 ^‡^

* All data are means ± standard error of means (SEM). Means within the same column followed by the same letter are not significantly different (*p* > 0.05). ^†^ ANOVA. ^‡^ Kruskal–Wallis test.

## Data Availability

Raw data are not publicly available but may be obtained upon request.
